# Supplementary material for: Resolving the full spectrum of human genome variation using Linked-Reads
Source: Genome Res. 2019 Apr;29(4):635–45. doi: 10.1101/gr.234443.118 (PMC6442396; doi:10.1101/gr.234443.118)
Supplement: Supplemental Material [file supp_gr.234443.118_Supplemental_longranger-2.2.zip › longranger-2.2.tar.bz2!/lr2.2-public/lib/stan/cmdstan/stan/doc/api/html/agrad_2rev_2functions_2exp2_8hpp_source.html]

Stan: src/stan/agrad/rev/functions/exp2.hpp Source File


|  |  |
| --- | --- |
|  | Stan  2.5.0  probability, sampling & optimization |


- Main Page
- Related Pages
- Namespaces
- Classes
- Files

- File List
- File Members

All Classes Namespaces Files Functions Variables Typedefs Enumerations Enumerator Friends Macros Pages

- src
- stan
- agrad
- rev
- functions

exp2.hpp

Go to the documentation of this file.

1 #ifndef STAN\_\_AGRAD\_\_REV\_\_FUNCTIONS\_\_EXP2\_HPP

2 #define STAN\_\_AGRAD\_\_REV\_\_FUNCTIONS\_\_EXP2\_HPP

3

4 #include <valarray>

5 #include <stan/agrad/rev/var.hpp>

6 #include <stan/agrad/rev/internal/v\_vari.hpp>

7 #include <stan/math/constants.hpp>

8 #include <math.h>

9

10 namespace stan {

11  namespace agrad {

12

13  namespace {

14  class exp2\_vari : public op\_v\_vari {

15  public:

16  exp2\_vari(vari\* avi) :

17  op\_v\_vari(::pow(2.0,avi->val\_),avi) {

18  }

19  void chain() {

20  avi\_->adj\_ += adj\_ \* val\_ \* stan::math::LOG\_2;

21  }

22  };

23  }

24

53  inline var exp2(const stan::agrad::var& a) {

54  return var(new exp2\_vari(a.vi\_));

55  }

56

57  }

58 }

59 #endif

stan::math::LOG\_2

const double LOG\_2

The natural logarithm of 2, .

**Definition:** constants.hpp:32

stan::agrad::exp2

fvar< T > exp2(const fvar< T > &x)

**Definition:** exp2.hpp:16

stan::agrad::pow

fvar< T > pow(const fvar< T > &x1, const fvar< T > &x2)

**Definition:** pow.hpp:17

stan::agrad::var::vi\_

vari \* vi\_

Pointer to the implementation of this variable.

**Definition:** var.hpp:40

v\_vari.hpp

var.hpp

stan::agrad::var

Independent (input) and dependent (output) variables for gradients.

**Definition:** var.hpp:27

constants.hpp


---

[ Stan Home Page ]

*© 2011–2014,
Stan Development Team.*
